# P05-18 The impact of a care-physical activity initiative for people with a low socioeconomic status on health, quality of life, and societal participation

**DOI:** 10.1093/eurpub/ckac095.085

**Published:** 2022-08-29

**Authors:** Lisanne Mulderij, Kirsten Verkooijen, Stef Groenewoud, Maria Koelen, Annemarie Wagemakers

**Affiliations:** 1 Social Sciences Department, Group Health and Society, Wageningen University & Research, Wageningen, The Netherlands; 2 Radboud Institute for Health Sciences, Scientific Centre for Quality of Healthcare, Radboud University Medical Centre, Nijmegen, The Netherlands

**Keywords:** Lifestyle intervention, Physical activity, Low socioeconomic status, Health promotion, Overweight and obesity

## Abstract

**Background:**

Overweight and obesity rates are increasing worldwide, particularly among people with a low socioeconomic status (SES). Care-physical activity (care-PA) initiatives may improve participants' lifestyles and thereby lower overweight and obesity rates. A two-year care-PA initiative specifically developed for citizens with a low SES, X-Fittt 2.0, was offered free of charge to participants, and included 12 weeks of intensive guidance and sports sessions, and 21 months of aftercare. Here, we study the impact of X-Fittt 2.0 on health, quality of life (QoL), and societal participation using a mixed-methods design.

**Methods:**

Questionnaires and body measurements were taken from 208 participants at the start of X-Fittt 2.0 (t0) and after 12 weeks (t1), one year (t2) and two to three years (t3). We also held 17 group discussions (t1, n = 71) and 68 semi-structured interviews (t2 and t3). Continuous variables were analysed using a linear mixed-model analysis (corrected for gender, age at t0, height, education level and employment status at the different time points), while we used descriptive statistics for the categorical variables. Qualitative data were analysed using a thematic analysis.

**Results:**

Body weight was significantly lower at all three post-initiative time points compared with the baseline, with a maximum of 3.8 kg difference at t2. BMI, waist circumference, blood pressure and self-perceived health only significantly improved during the first 12 weeks. A positive trend regarding paid work was observed, while social visits decreased. The latter might be explained by the COVID-19 pandemic, as lockdowns limited social life. Furthermore, participants reported increased PA (including sports) and a few stopped smoking or drinking alcohol. Participants mentioned feeling healthier, fitter and more energetic. Additionally, participants' self-esteem and stress levels improved, stimulating them to become more socially active. However, the participants also mentioned barriers to being physically active, such as a lack of money or time, or physical or mental health problems.

**Conclusions:**

X-Fittt 2.0 improved the health, QoL and societal participation of the participants. Future initiatives should take into account the aforementioned barriers, and consider a longer intervention period for more sustainable results. More complete data are needed to confirm the findings.

